# Urokinase-type plasminogen activator and arthritis progression: contrasting roles in systemic and monoarticular arthritis models

**DOI:** 10.1186/ar3171

**Published:** 2010-10-25

**Authors:** Christine M De Nardo, Jason C Lenzo, Jarrad Pobjoy, John A Hamilton, Andrew D Cook

**Affiliations:** 1 Arthritis and Inflammation Research Centre, Department of Medicine, The University of Melbourne, Royal Parade, Parkville, Melbourne, Victoria 3010, Australia

## Abstract

**Introduction:**

Urokinase-type plasminogen activator (u-PA) has been implicated in tissue destruction/remodeling. The absence of u-PA results in resistance of mice to systemic immune complex-driven arthritis models; monoarticular arthritis models involving an intra-articular (i.a.) antigen injection, on the other hand, develop more severe arthritis in its absence. The aims of the current study are to investigate further these contrasting roles that u-PA can play in the pathogenesis of inflammatory arthritis and to determine whether u-PA is required for the cartilage and bone destruction associated with disease progression.

**Methods:**

To determine how the different pathogenic mechanisms leading to arthritis development in the different models may explain the contrasting requirement for u-PA, the systemic, polyarticular, immune complex-driven K/BxN arthritis model was modified to include an i.a. injection of saline as a local trauma in u-PA^-/- ^mice. This modified model and the antigen-induced arthritis (AIA) model were also used in u-PA^-/- ^mice to determine the requirement for u-PA in joint destruction. Disease severity was determined by clinical and histologic scoring. Fibrin(ogen) staining and the matrix metalloproteinase (MMP)-generated neoepitope DIPEN staining were performed by immunohistochemistry. Gene expression of inflammatory and destructive mediators was measured in joint tissue by quantitative PCR.

**Results:**

In our modified arthritis model, u-PA^-/- ^mice went from being resistant to arthritis development following K/BxN serum transfer to being susceptible following the addition of an i.a. injection of saline. u-PA^-/- ^mice also developed more sustained AIA compared with C57BL/6 mice, including reduced proteoglycan levels and increased bone erosions, fibrin(ogen) deposition and DIPEN expression. Synovial gene expression of the proinflammatory mediators (TNF and IL-1β), aggrecanases (ADAMTS-4 and -5) and MMPs (MMP3 and MMP13) were all sustained over time following AIA induction in u-PA^-/- ^mice compared with C57BL/6 mice.

**Conclusions:**

We propose that u-PA has a protective role in arthritis models with 'wound healing-like' processes following local trauma, possibly through u-PA/plasmin-mediated fibrinolysis, but a deleterious role in systemic models that are critically dependent on immune complex formation and complement activation. Given that cartilage proteoglycan loss and bone erosions were present and sustained in u-PA^-/- ^mice with monoarticular arthritis, it is unlikely that u-PA/plasmin-mediated proteolysis is contributing directly to this tissue destruction/remodeling.

## Introduction

Urokinase-type plasminogen activator (u-PA) is a serine protease that cleaves plasminogen to form plasmin [1]. The u-PA/plasmin system has been implicated in a number of processes, including fibrinolysis, cell migration, cell activation, and tissue remodeling (directly or indirectly via matrix metalloproteinase [MMP] activation) (reviewed in [1,2]). The systemic polyarthritic collagen-induced arthritis (CIA), type II collagen monoclonal antibody-induced arthritis (CAIA), and K/BxN serum transfer arthritis models have all been reported to be dependent on u-PA for full development of disease [3-5]. These models are all immune complex-mediated and complement-dependent, and we have previously suggested that u-PA involvement may be upstream of C5a signaling [4]. However, as most u-PA^-/- ^mice do not develop any significant disease in these models, they are not the best models for determining whether u-PA is required for many of the processes described above, such as tissue remodeling, as they do not come into play to any extent because of the minor inflammatory reaction in the joints. In contrast to the findings with the systemic models, the monoarticular arthritis models - namely antigen-induced arthritis (AIA) [6] and methylated bovine serum albumin/interleukin-1 (mBSA/IL-1) arthritis [7], both of which use mBSA as the antigen and involve an intra-articular (i.a.) injection - develop more severe disease in the absence of u-PA. These latter models therefore should be ideal to determine whether u-PA can have a role in tissue destruction/remodeling, for example.

Joint damage in arthritis, with cartilage and bone destruction, is believed to be mediated (at least in part) through proteases, such as MMPs and plasmin [8]. u-PA-generated plasmin activity may play a pivotal role in this degradative process, either indirectly through the activation of latent MMPs or directly through the ability of plasmin to degrade cartilage proteoglycans as well as other cartilage and bone matrix proteins [2,9,10]. Several different cell types present in arthritic joints can produce PAs and their inhibitors *in vitro*, including in response to inflammatory cytokines [11-18]. Furthermore, in different *in vitro *experimental models, u-PA, derived from the tissue cells in question, has been shown to contribute to cartilage and bone destruction [19,20]. In the monoarticular arthritis models, AIA [6] and mBSA/IL-1 [7], using u-PA^-/- ^mice, fibrin deposition appeared to parallel disease severity, suggesting that u-PA-mediated fibrinolysis normally may play a protective role in inflammatory joint disease. No evidence was found in either model for u-PA to be required for cell migration; in fact, increased cellular infiltration was seen in injected joints of the u-PA^-/- ^mice. In regard to joint damage, in the more severe AIA model, u-PA^-/- ^mice were observed to have increased bone erosion; however, the extent of cartilage damage was no different from that seen in wild-type mice [6].

To determine how the different pathogenic mechanisms leading to arthritis development in the different models (systemic immune complex-driven versus local trauma induced by i.a. injection) may explain the contrasting requirement for u-PA (as was found for plasminogen using the CIA model with an i.a. injection of type II collagen [21]), we have used a similar approach, combining the K/BxN immune complex-driven arthritis model with an i.a. injection of saline. u-PA^-/- ^mice were resistant to the systemic arthritis induction but developed arthritis, including proteoglycan loss, in the joint that received an i.a. injection of saline. Given this result, we re-examined whether lack of u-PA could lead to enhanced cartilage proteoglycan loss in the AIA model. These mice developed a more sustained arthritis, including reduced proteoglycan levels and increased fibrin deposition, indicating that u-PA is not required for cartilage and bone destruction but actually plays a protective role in AIA, possibly because of its fibrinolytic activity.

## Materials and methods

### Mice

u-PA gene-deficient (u-PA^-/-^) mice, originally provided by Professor Peter Carmeliet (University of Leuven, Leuven, Belgium), were backcrossed onto the C57BL/6 background for 11 generations. The two strains then were bred separately, and the C57BL/6 strain was reintroduced to the u-PA^-/- ^strain every four to five generations. All strains were bred in our on-site animal facility, fed standard rodent chow and water *ad libitum*, and housed in sawdust-lined cages in groups of five. Mice of both sexes, 8 to 12 weeks of age, were used in all experiments. All experiments were approved by the University of Melbourne Animal Ethics Committee.

### K/BxN serum transfer model of arthritis with intra-articular injection of saline

K/BxN mice were bred and serum was collected as described previously [22]. Serum was collected up to 12 weeks of age and stored at -80°C. K/BxN serum transfer arthritis was induced in C57BL/6 mice and u-PA^-/- ^mice as before [4]. On day 0, mice also received an i.a. injection of 10 μL of sterile saline or were left untreated. Mice were scored daily as before [4]. On day 10 following serum transfer, mice were sacrificed and mouse knees were given a 'macroscopic knee score' ranging from 0 to 3: 0, knee joint shows no signs of inflammation; 1, knee is mildly swollen; 2, knee swelling is severe with flexibility; 3, knee swelling is severe and loss of flexibility (ankylosis). Knee joints were collected for histological analysis.

### Antigen-induced arthritis

Mice were immunized on days 0 and 7 with 100 μg of mBSA (Sigma-Aldrich, Buchs, Switzerland), emulsified in complete Freund's adjuvant containing 200 μg of *Mycobacterium tuberculosis *H37RA (Difco, now part of Becton Dickinson and Company, Franklin Lakes, NJ, USA), by a 0.1-mL intra-dermal injection at the base of the tail. Arthritis was induced at day 21 by an i.a. injection of 100 μg of mBSA in 10 μL of sterile phosphate-buffered saline (PBS) into the right knee, and the left knee was injected with sterile PBS. Mice were sacrificed at weeks 1, 2, and 6 following i.a. injection; mouse knees were given a 'macroscopic knee score' ranging from 0 to 3 as described above, and knee joints were collected for histological analysis and synovial tissue was collected for mRNA analysis.

### Histology

At termination following arthritis induction, the knee joints were removed, fixed, decalcified, and paraffin-embedded, as previously described [3]. Frontal sections (5 μm) were stained either with hematoxylin and eosin to examine joint architecture or with safranin O, fast green, and hematoxylin for proteoglycan loss, and evaluated without knowledge of the experimental groups by using the histologic assessment as published [3,4]. Briefly, infiltration of cells and cartilage damage were all scored separately from 0 (normal) to 3 (severe), and bone erosions were scored from 0 (normal) to 4 (severe); proteoglycan loss (safranin O, fast green stain) was scored from 0 (normal) to 3 (complete loss of staining).

### Immunohistochemistry

Fibrin(ogen) deposition was identified in knee joints by using a goat anti-mouse fibrinogen/fibrin antibody (Accurate Chemical & Scientific Corporation, Westbury, NY, USA), as before [4]. The MMP-induced neoepitope, DIPEN, was detected as published [23] but with slight modifications. Briefly, paraffin-embedded sections were deparaffinized and rehydrated, and endogeneous peroxidise was blocked with 3% (vol/vol) H_2_O_2 _(Sigma-Aldrich). Sections were digested with chondroitinase ABC (0.1 units/mL; Sigma-Aldrich) for 2 hours at 37°C to remove chondroitin sulfate from the proteoglycans, prior to blocking with 5% normal goat serum. Sections then were incubated overnight at 4°C with anti-DIPEN [24] (a gift from Assistant Professor Amanda J Fosang, University of Melbourne, Victoria, Australia) and detected with a biotinylated anti-rabbit IgG (Dako, Glostrup, Denmark) followed by a streptavidin-peroxidase conjugate (BD Biosciences, San Jose, CA, USA). Peroxidase activity was demonstrated by incubation with DAB (3,3'-diaminobenzidine/tetrahydrochloride) (Sigma-Aldrich)-H_2_O_2 _solution. Slides were counterstained with hematoxylin.

### Quantitative polymerase chain reaction analysis of gene expression

Quantitative polymerase chain reaction (qPCR) was performed as before [4]. Briefly, the synovium from knee joints was removed, RNA was extracted using the RNeasy Mini Kit (Qiagen, Valencia, CA, USA), and cDNA was prepared. qPCR was performed using Predeveloped TaqMan gene expression assays for tumor necrosis factor-alpha (TNFα), IL-1β, ADAMTS-4 (a disintegrin and metalloproteinase with thrombospondin motifs 4), ADAMTS-5, MMP-3, and MMP-13 (Applied Biosystems, Foster City, CA, USA) and read on an ABI Prism 7900H sequence detection system followed by analysis with ABI Prism SDS 2.1 software. The TATA-binding protein (GeneWorks, Hindmarsh, SA, Australia) was used as the control gene. The comparative threshold method for relative quantification was used, and results are expressed as relative gene expression for each target gene.

### Statistics

For clinical and histologic scores and gene expression studies, the Mann-Whitney two-sample rank test was used to determine the level of significance between strains or treatment groups, and the Kruskal-Wallis rank sum test was used to determine the level of significance over time in the AIA model; values are expressed as the mean ± standard error of the mean. A *P *value of not more than 0.05 was considered statistically significant.

## Results

### Increased arthritis in an injected joint of a u-PA^-/- ^mouse with K/BxN serum transfer arthritis

As discussed above, previous studies in u-PA^-/- ^mice have shown opposite results in regard to the effects of u-PA on disease progression in different arthritis models [3-7]. These can be divided into (a) systemic polyarticular models (CIA, CAIA, and K/BxN serum transfer [3-5]) involving immune complex deposition in the joint, where the presence of u-PA is deleterious, and (b) monoarticular models (AIA and mBSA/IL-1 [6,7]) involving an i.a. injection of mBSA into the joint, where it is protective (Table 1). While the models differ in the antigens recognized, they also differ in the manner in which the arthritis is induced. In the polyarticular models, an intradermal, intraperitoneal, or intravenous injection leads to systemic disease, whereas the monoarticular models involve an i.a. injection of antigen (for example, the cationic protein, mBSA), leading to local trauma in the knee joint. If the mouse has been previously primed with the antigen (as in the AIA model) or given a systemic stimulus such as IL-1β (as in the mBSA/IL-1 arthritis model), then arthritis develops in the injected joint. However, if mice are given an i.a. injection of saline only, then 7 days after i.a. injection, the joints appear normal, even when IL-1β is given subcutaneously [25]. To determine how the different elicitation protocols may explain these opposite results in u-PA^-/- ^mice, we used an approach similar to that of Li and colleagues [21] but combined the systemic K/BxN arthritis model with an i.a. injection of only saline.

**Table 1:** Comparison of arthritis development in u-PA^-/- ^mice in different arthritis models

	Polyarticular u-PA is deleterious^a^	Monoarticular u-PA is protective^b^
Induction	Systemic (intra-dermal, intravenous, and intraperitoneal)	Intra-articular
Models	CIA [3,5], CAIA [4], and K/BxN serum transfer [4]	mBSA/IL-1 [7] and AIA [6]
Mechanism of arthritis development	Immune complex-mediated and complement-dependent	Local trauma, increased fibrin deposition, and 'wound healing-like' process

^a^u-PA is deleterious; that is, disease was reduced in u-PA^-/- ^mice. ^b^u-PA is protective; that is, disease was worse in u-PA^-/- ^mice. AIA, antigen induced arthritis; CAIA, type II collagen monoclonal antibody-induced arthritis; CIA, collagen-induced arthritis; mBSA, methylated bovine serum albumin; u-PA, urokinase-type plasminogen activator; u-PA^-/-^, urokinase-type plasminogen activator gene-deficient.

**Figure 1:**
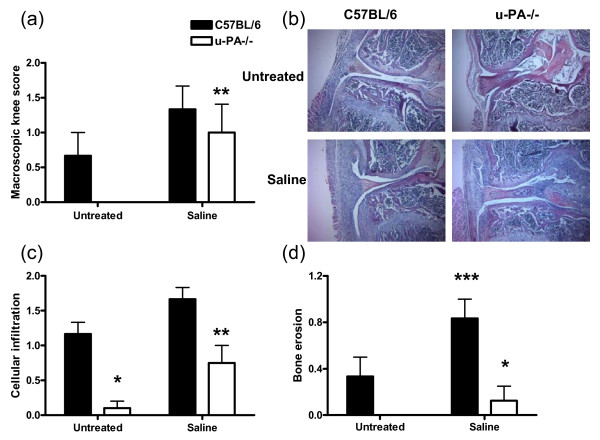
**u-PA**^-/- ^**mice are resistant to K/BxN serum transfer arthritis but display monoarticular arthritis following the addition of an intra-articular injection**. C57BL/6 and u-PA^-/- ^mice with K/BxN serum transfer arthritis were either left untreated or given an intra-articular injection of saline. Mice were sacrificed 10 days later. **(a) **Macroscopic knee score. **(b) **Representative histological pictures (hematoxylin and eosin) of knee joints. Quantification of **(c) **cellular infiltration and **(d) **bone erosions in knee joints. Results are expressed as the mean ± standard error of the mean (five mice per group). *P = 0.002, u-PA^-/- ^versus C57BL/6 mice; **P = 0.002, ***P < 0.05, saline-treated versus untreated mice. u-PA^-/-^, urokinase-type plasminogen activator gene-deficient.

As reported before [4], u-PA^-/- ^mice were essentially resistant to the K/BxN serum transfer arthritis model (data not shown). As judged by swelling (macroscopic knee score), their knee joints appeared normal (Figure 1a, 'untreated' group); by contrast, the knee joints of C57BL/6 mice, which are susceptible to K/BxN serum transfer arthritis [4], showed some swelling, indicative of inflammation (Figure 1a, 'untreated' group). However, when (in addition to the K/BxN serum) mice received an i.a. injection of saline, both C57BL/6 and u-PA^-/- ^mice showed increased knee joint swelling compared with that found in untreated (non-injected) knees (Figure 1a). Given that there was no joint swelling in the untreated knees of u-PA^-/- ^mice, this increased swelling seen following the i.a. injection was relatively greater than in C57BL/6 mice, in which a degree of joint swelling was already present (Figure 1a).

By histology, at 10 days after K/BxN serum transfer, the untreated knee joints of C57BL/6 mice showed inflammatory cell infiltration and bone erosions, which were absent from the knee joints of u-PA^-/- ^mice (Figure 1b-d). However, in the saline-treated C57BL/6 and u-PA^-/- ^knee joints, cellular infiltration and bone erosions were increased compared with the respective untreated knee joints (Figure 1b-d). The degree of bone erosion present in u-PA^-/- ^mice was lower than in C57BL/6 mice following i.a. injection of saline (*P *< 0.05) (Figure 1d). However, once again, the baseline levels of both cellular infiltration and bone erosions in the untreated knee joints of u-PA^-/- ^mice were lower than in the untreated knee joint of C57BL/6 mice, and this may account for the relative differences seen between the strains following i.a. injection.

**Figure 2:**
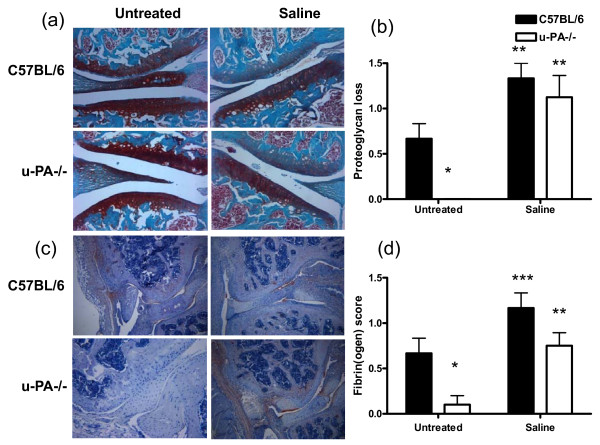
**After the addition of an intra-articular injection, u-PA**^-/- ^**mice with K/BxN serum transfer arthritis show proteoglycan loss and fibrin(ogen) staining in the injected knee joints only**. C57BL/6 and u-PA^-/- ^mice with K/BxN serum transfer arthritis were either left untreated or given an intra-articular injection of saline. Mice were sacrificed 10 days later. **(a) **Representative histological staining of knee joints for proteoglycan (safranin O) and **(b) **quantification of proteoglycan loss. **(c) **Representative histological staining of knee joints for fibrin(ogen) staining and **(d) **quantification of fibrin(ogen) staining. Results are expressed as the mean ± standard error of the mean (five mice per group). *P = 0.002, untreated u-PA^-/- ^versus C57BL/6 mice; **P < 0.01, ***P < 0.05, saline-treated versus untreated mice. u-PA^-/-^, urokinase-type plasminogen activator gene-deficient.

For proteoglycan content, untreated knee joints from C57BL/6 mice with K/BxN serum transfer arthritis showed some loss 10 days following serum transfer, whereas similarly treated u-PA^-/- ^mice demonstrated intact cartilage with no proteoglycan loss (*P *= 0.002; Figure 2a, b). Following the i.a. injection of saline, u-PA^-/- ^mouse knee joints demonstrated a decrease in proteoglycan content to the same extent as that seen in C57BL/6 mice (Figure 2a, b). However, relative to the corresponding untreated knee joints, u-PA^-/- ^mice showed a greater increase in proteoglycan loss following i.a. injection compared with that for C57BL/6 mice.

Consistent with all histological parameters analyzed above, fibrin(ogen) staining was significantly greater in the untreated knee joints of C57BL/6 mice with K/BxN serum transfer arthritis compared with similarly treated u-PA^-/- ^mice (*P *= 0.002; Figure 2c, d). Following i.a. injection of saline, comparable amounts of fibrin(ogen) were found in C57BL/6 and u-PA^-/- ^knee joints (Figure 2c, d). Once again, relative to the corresponding untreated knee joints, u-PA^-/- ^mice showed a greater increase in fibrin(ogen) deposition following i.a. injection compared with that for C57BL/6 mice.

Thus, following an i.a injection of saline, K/BxN serum transfer-treated u-PA^-/- ^mice developed arthritis in the injected joint but in no other joints. The degree of cellular infiltration, cartilage damage, bone erosions, and fibrin(ogen) staining was increased, relative to the values in untreated joints, in u-PA^-/- ^mice and C57BL/6 mice following the i.a. injection, and the resultant levels often were similar between the two strains, despite the higher 'baseline' levels in C57BL/6 mice prior to the injection.

### Increased arthritis in u-PA^-/- ^mice in the antigen-induced arthritis model

For the systemic K/BxN serum transfer model, the reduced cartilage proteoglycan loss in u-PA^-/- ^mice is consistent with the involvement of plasmin-mediated proteolytic activity in this tissue destruction, either directly or indirectly via MMP activation [9,10]. The higher relative cartilage proteoglycan loss found above in this model following i.a. injection of saline with u-PA^-/- ^mice indicates that, following such trauma, the presence of u-PA is in fact protective of such loss, noting that the relative baseline levels ('untreated') of proteoglycan differed between the two strains. In the monoarticular AIA model involving i.a. antigen challenge, however, Busso and colleagues [6] did not find any difference in cartilage proteoglycan depletion between u-PA^-/- ^mice and their corresponding wild-type controls, even though the arthritis was more severe in the former mice. We therefore re-examined whether lack of u-PA could lead to enhanced cartilage proteoglycan loss in the more severe AIA model.

### Cartilage damage and bone erosions

**Figure 3:**
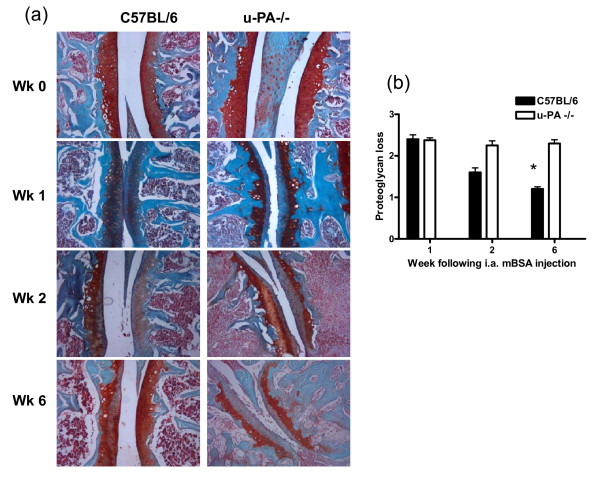
**u-PA**^-/- ^**mice show sustained proteoglycan loss in their knee joints following antigen-induced arthritis induction**. Mice were sacrificed at 0, 1, 2, and 6 weeks after intra-articular (i.a.) injection. **(a) **Representative proteoglycan staining (safranin O). **(b) **Quantification of proteoglycan loss. Results are expressed as the mean ± standard error of the mean (five mice per group). *P = 0.008, C57BL/6 versus u-PA^-/- ^mice; *P *= 0.015, C57BL/6 over time. mBSA, methylated bovine serum albumin; u-PA^-/-^, urokinase-type plasminogen activator gene-deficient.

To confirm whether a lack of u-PA had any effect on cartilage matrix, the proteoglycan content throughout the course of AIA development over a 6-week period was compared in C57BL/6 and u-PA^-/- ^mice (Figure 3a, b). Induction of AIA led to a significant decrease in proteoglycan content, as demonstrated by the loss of safranin O staining, at 1 week after i.a. injection in both C57BL/6 and u-PA^-/- ^mouse knee joints (Figure 3a, b). Throughout the remaining 5 weeks, the proteoglycan content increased in C57BL/6 mice, indicating recovery (*P *= 0.015, proteoglycan content of C57BL/6 mice over time; Figure 3a, b); in contrast, the proteoglycan content was not restored in u-PA^-/- ^mice, which displayed significantly less proteoglycan staining at week 6 compared with C57BL/6 mice (*P *= 0.008; Figure 3b). Thus, in contrast to the results of Busso and colleagues [6], a restoration of proteoglycan was seen following the initial loss at week 1 in C57BL/6 mice but there was no such recovery in u-PA^-/- ^mice.

**Figure 4:**
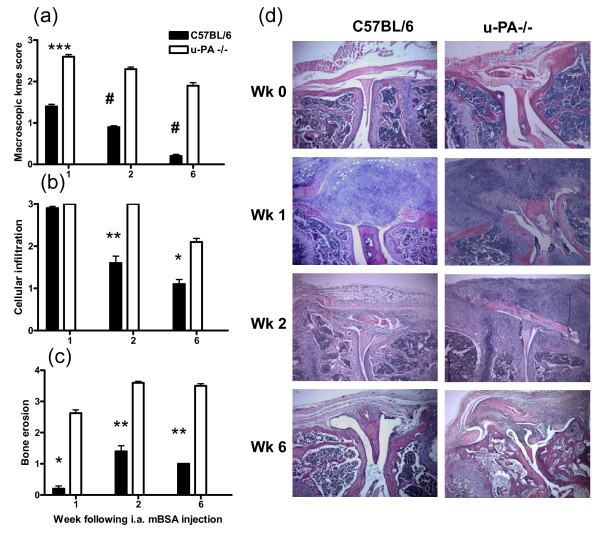
**u-PA**^-/- ^**mice develop increased antigen-induced arthritis**. Mice were sacrificed at 0, 1, 2, and 6 weeks after intra-articular (i.a.) injection. **(a) **Macroscopic knee score. Quantification of **(b) **cellular infiltration and **(c) **bone erosions in knee joints. **(d) **Representative histological pictures (hematoxylin and eosin) of knee joints. Results are expressed as the mean ± standard error of the mean (five mice per group). *P < 0.05, **P < 0.01, ***P < 0.001, ^#^*P *< 0.0001, C57BL/6 versus u-PA^-/- ^mice; *P *= 0.0001, C57BL/6 macroscopic knee score over time; *P *= 0.007, C57BL/6 cellular infiltration over time; *P *< 0.05, C57BL/6 or u-PA^-/- ^bone erosion over time. mBSA, methylated bovine serum albumin; u-PA^-/-^, urokinase-type plasminogen activator gene-deficient.

This result of sustained proteoglycan loss in u-PA^-/- ^mice following AIA induction compared with C57BL/6 mice was mirrored both in the degree of knee swelling seen over time (Figure 4a) and in the degree of cellular infiltration into the joint (Figure 4b, d). u-PA^-/- ^mice also showed increased and sustained bone erosion compared with C57BL/6 mice (Figure 4c, d). There was also significantly greater fibrin(ogen) deposition in u-PA^-/- ^mouse joints following AIA induction compared with that found in C57BL/6 mice at each time point (1, 2, and 6 weeks) (data not shown).

### Synovial gene expression of inflammatory and destructive mediators

**Figure 5:**
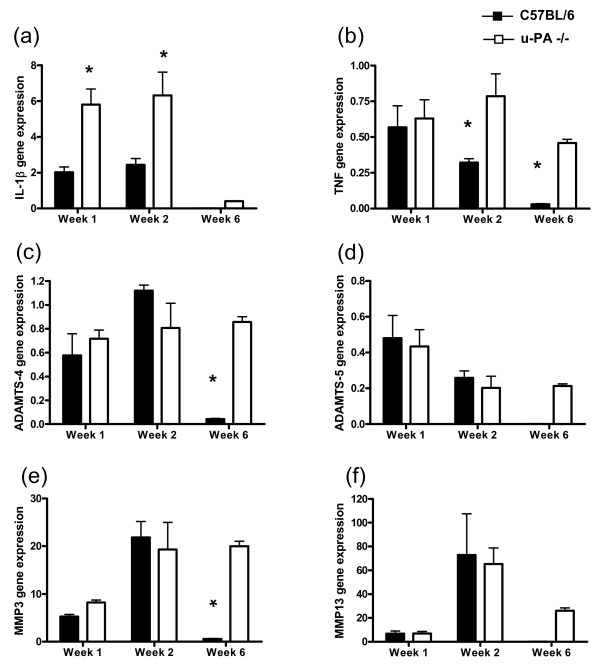
**u-PA**^-/- ^**mice show sustained synovial gene expression of inflammatory and destructive mediators during antigen-induced arthritis**. Relative mRNA gene expression in the synovial tissue from u-PA^-/- ^and C57BL/6 mice at 1, 2, and 6 weeks after intra-articular injection in the antigen-induced arthritis model. **(a) **Interleukin-1-beta (IL-1β). **(b) **Tumor necrosis factor (TNF). **(c) **ADAMTS-4. **(d) **ADAMTS-5. **(e) **Matrix metalloproteinase-3 (MMP-3). **(f) **MMP-13. Results are expressed as the mean ± standard error of the mean (five mice per group). *P < 0.01, C57BL/6 versus u-PA^-/- ^mice; *P *< 0.05, C57BL/6 IL-1β, TNF, MMP-13 over time, *P *= 0.01, C57BL/6 ADAMTS-4, MMP-3 over time; *P *< 0.01, u-PA^-/- ^MMP-13 over time. IL-1β, ADAMTS-5, and MMP-13 were not detectable in C57BL/6 mice at week 6. ADAMTS, a disintegrin and metalloproteinase with thrombospondin motifs; u-PA^-/-^, urokinase-type plasminogen activator gene-deficient.

To gain some insight into the possible mechanisms accounting for the sustained AIA severity and proteoglycan loss in u-PA^-/- ^mice, we determined whether there was increased or sustained (or both) synovial tissue expression of genes involved in inflammation and cartilage degradation. In regard to proinflammatory cytokines, the increased synovial tissue IL-1β (Figure 5a) and TNF (Figure 5b) mRNA expression noted in C57BL/6 mice early in disease decreased to low levels, in line with the degree of swelling (Figure 4a), but was sustained in the u-PA^-/- ^mice. A similar pattern was observed for the aggrecanases, ADAMTS-4 (Figure 5c) and ADAMTS-5 (Figure 5d), implicated in cartilage proteoglycan breakdown [26], and for MMP-3 (stromelysin-1) (Figure 5e) and MMP-13 (collagenase-3) (Figure 5f), implicated in the breakdown of extracellular matrices, including proteoglycan [26-30].

### Expression of a proteoglycan neoepitope

**Figure 6:**
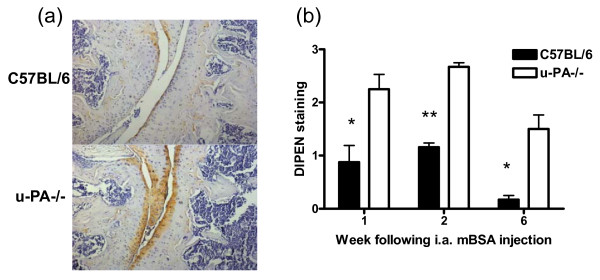
**u-PA**^-/- ^**mice show increased expression of the matrix metalloproteinase-mediated neoepitope, DIPEN, during antigen-induced arthritis**. **(a) **Representative DIPEN staining in knee joints from C57BL/6 and u-PA^-/- ^mice at week 1 after intra-articular (i.a.) injection. **(b) **Quantification of DIPEN staining at weeks 1, 2, and 6 after i.a. injection. Results are expressed as the mean ± standard error of the mean (five mice per group). *P = 0.03, **P = 0.003, C57BL/6 versus u-PA^-/- ^mice; *P *= 0.003, C57BL/6 over time; *P *= 0.03, u-PA^-/- ^over time. mBSA, methylated bovine serum albumin; u-PA^-/-^, urokinase-type plasminogen activator gene-deficient.

Given the above expression of key MMPs in the joints of both C57BL/6 and u-PA^-/- ^mice following AIA induction (combined with loss of proteoglycan), expression of the MMP-generated proteoglycan neoepitope, DIPEN, was assessed by immunohistochemistry at 1, 2, and 6 weeks after AIA induction. In C57BL/6 and u-PA^-/- ^mice, DIPEN staining in the cartilage was present at 1 week (Figure 6a). In C57BL/6 mice, this staining was still evident at 2 weeks, with less staining evident by 6 weeks (Figure 6b), in line with the view that proteoglycan loss is due to increased cartilage destruction; u-PA^-/- ^mice showed increased and sustained DIPEN staining compared with C57BL/6 mice (Figure 6b), once again in line with continuing proteoglycan destruction.

## Discussion

It has been previously demonstrated using u-PA^-/- ^mice in three systemic arthritis models - namely the CIA, CAIA, and K/BxN serum transfer models - that when u-PA was absent, disease was reduced [3-5]; the similar data for both the CIA and CAIA models in plasminogen-deficient mice [5] are consistent with the involvement of u-PA/plasmin-mediated proteolysis. The proteolytic activity of the u-PA/plasmin system itself or via MMP activation could be contributing directly to tissue (cartilage and bone) destruction/remodeling, especially since there are *in vitro *data showing that increased plasmin activity can enhance cartilage breakdown [19,20,29]. Paradoxically, in the two monoarticular models involving i.a. antigen injection - namely the AIA and mBSA/IL-1 models - when u-PA was absent, disease activity was exacerbated [6,7]. It is likely that this protective effect of u-PA is again due to u-PA/plasmin-mediated proteolysis since similar data were reported for the AIA model in plasminogen-deficient mice [6]. Interestingly, direct i.a. injection of low-molecular-weight human u-PA has been reported to induce arthritis in mice [31]. In this case, however, plasminogen was not the major mediator of the u-PA-induced inflammation.

To attempt to clarify the role of u-PA in arthritis progression, we have modified one of the systemic models - namely the K/BxN serum transfer model - by incorporating an i.a. injection of saline to induce some trauma into a knee joint or, in other words, to combine aspects of the systemic and monoarticular models in u-PA^-/- ^mice; a similar approach by Li and colleagues [21] showed that the joint trauma caused by a local i.a. injection of type II collagen into CIA-immunized plasminogen^-/- ^mice, which are normally resistant to arthritis development [5], led to arthritis in the injected joint. In the present study, we found a similar result in the sense that u-PA^-/- ^mice went from being resistant to arthritis development following K/BxN serum transfer to being susceptible following the addition of an i.a. injection of saline. This arthritis development following i.a. injection of only saline, which by itself does not lead to arthritis [25], could be due to the prior presence of the GPI (glucose-6-phosphate isomerase) antibodies and their immune complexes in the injected joint (from the K/BxN serum transfer [32]), contributing to the prolongation of the normally acute inflammatory response associated with the trauma of the injection and leading subsequently to arthritis development in that joint. Since cartilage proteoglycan loss and bone erosions were now observed in the joint receiving the i.a. injection in the absence of u-PA, it makes it unlikely, in our view, that u-PA/plasmin-mediated proteolysis, either itself or via MMP activation [9,10], is contributing directly to this tissue destruction/remodeling. However, it cannot be excluded that, in u-PA^-/- ^mice, the absence of u-PA may be compensated for by the activities of one or more other pathways or proteinases or both. Data from this new model are similar to what we found in the AIA model (Figures 3-6); and, as proposed before [6,33,34], u-PA/plasmin may be protective following trauma by removing unwanted material (for example, fibrin). While tissue-type plasminogen activator (t-PA)^-/- ^mice display more severe arthritis in both monoarticular and polyarticular models, attributable to increased fibrin deposition in the joint [3,7], in models incorporating an i.a. injection, u-PA may also play a role in such fibrinolysis [2,6]. The increased fibrin deposition, relative to baseline ('untreated'), seen in the joints of u-PA^-/- ^mice receiving an i.a. injection (K/BxN serum transfer model with an i.a. injection [Figure 2] and in joints in the AIA model [6]), supports this conclusion and may have led to the ongoing inflammatory response. Fibrin(ogen) deposition within joints has been shown to contribute to arthritic disease development via Mac-1-dependent leukocyte activation programs, including the expression of proinflammatory cytokines, but is not strictly required for CIA development [35]. However, arthritic disease driven by exuberant TNFα expression, for example, was fibrin(ogen)-independent [35]. It is worth noting that there is evidence that u-PA is required for skeletal muscle regeneration [36] and has been implicated in myocardial infarct repair [37].

Following on from the above observations in our modified systemic model, in the more severe monoarticular AIA model, we not only found that an absence of u-PA led to sustained inflammation and bone destruction (as previously reported [6]) but also showed that proteoglycan content returned over time in C57BL/6 mice but did not in u-PA^-/- ^mice. This was coupled with increased and prolonged expression of the MMP-generated neoepitope DIPEN 6 weeks after i.a. injection in the absence of u-PA. Likewise, synovial gene expression of major proinflammatory mediators (IL-1β and TNF), aggrecanases (ADAMTS-4 and -5), and the MMPs (MMP-3 and -13) showed continued expression in the absence of u-PA, whereas in its presence, their expression had fallen to negligible levels, reflecting disease severity. The above gene changes may be linked since higher IL-1β and TNF levels may in turn upregulate ADAMTS-4 [38] and the MMPs [39,40], as well as contribute to inhibition of cartilage matrix synthesis [38,41] and increased bone resorption [42,43].

Previous studies from our laboratory have shown that, in the systemic CIA model, a bone marrow-derived cell or cells, presumably following migration into the inflamed synovium, produce the u-PA required for optimal disease severity [4]. We have previously observed a reduced myeloid cell infiltrate in u-PA^-/- ^mice following only immune complex-mediated peritonitis [4] and not in response to two other stimuli, including mBSA [44]. From the present study, sustained cell infiltration and cartilage proteoglycan loss occurred in u-PA^-/- ^mice following i.a. injection in two monoarticular models, suggesting that u-PA in the inflamed joint is not involved directly in these aspects *per se*. Given that (in the absence of u-PA or plasminogen) systemic immune complex-mediated arthritis models are resistant to full disease development [3-5], it could be that synovial u-PA/plasmin is a critical component of an immune complex-mediated inflammatory reaction, perhaps indirectly by activating complement (C5a) [4], leading to cellular infiltration (as suggested previously [4,5,21]), rather than being involved directly in cell migration or tissue damage. Unlike immune complex-mediated inflammatory reactions, there is evidence that the complement system is not a primary mediator of the inflammation following a wound or trauma, such as in the case of an i.a. injection, in which neutrophils can be activated in the absence of active complement [45].

## Conclusions

The results of these and other studies [3-7] highlight the contrasting roles that u-PA can play in the pathogenesis of inflammatory arthritis. In summary, we propose that u-PA has a protective role in arthritis models with 'wound healing-like' processes following local trauma, possibly through u-PA/plasmin mediated fibrinolysis, but a deleterious role in systemic models that are critically dependent on immune complex formation and complement activation. In regard to human arthritis, u-PA blockade therefore may be a therapeutic strategy for systemic, immune complex-mediated conditions, such as rheumatoid arthritis, but not for those that may result from joint injury or trauma.

## Abbreviations

ADAMTS: a disintegrin and metalloproteinase with thrombospondin motifs; AIA: antigen-induced arthritis; CAIA: type II collagen monoclonal antibody-induced arthritis; CIA: collagen-induced arthritis; i.a.: intra-articular; IL: interleukin; mBSA: methylated bovine serum albumin; MMP: matrix metalloproteinase; PBS: phosphate-buffered saline; qPCR: quantitative polymerase chain reaction; TNF: tumor necrosis factor; u-PA: urokinase-type plasminogen activator; u-PA^-/-^: urokinase-type plasminogen activator gene-deficient.

## Competing interests

The authors declare that they have no competing interests.

## Authors' contributions

CMDN performed all of the arthritis experiments and was involved in all aspects of the study. JCL participated in the immunohistochemistry. JP was involved in generating the gene expression data. JAH conceived the study, participated in its design, and helped to draft the manuscript. ADC conceived the study, participated in its design and coordination, and drafted the manuscript. All authors read and approved the final manuscript.

## References

[B1] Irigoyen JP Munoz-Canoves P Montero L Koziczak M Nagamine Y The plasminogen activator system: biology and regulation Cell Mol Life Sci 1999 56 104 132 10.1007/PL00000615 11213252 PMC11146966

[B2] Busso N Hamilton JA Extravascular coagulation and the plasminogen activator/plasmin system in rheumatoid arthritis Arthritis Rheum 2002 46 2268 2279 10.1002/art.10498 12355473

[B3] Cook AD Braine EL Campbell IK Hamilton JA Differing roles for urokinase and tissue-type plasminogen activator in collagen-induced arthritis Am J Pathol 2002 160 917 926 11891190 10.1016/S0002-9440(10)64914-0 PMC1867189

[B4] Cook AD De Nardo CM Braine EL Turner AL Vlahos R Way KJ Beckman SK Lenzo JC Hamilton JA Urokinase-type plasminogen activator and arthritis progression: role in systemic disease with immune complex involvement Arthritis Res Ther 2010 12 R37 10.1186/ar2946 20196869 PMC2888184

[B5] Li J Ny A Leonardsson G Nandakumar KS Holmdahl R Ny T The plasminogen activator/plasmin system is essential for development of the joint inflammatory phase of collagen type II-induced arthritis Am J Pathol 2005 166 783 792 15743790 10.1016/S0002-9440(10)62299-7 PMC1602367

[B6] Busso N Peclat V Van Ness K Kolodziesczyk E Degen J Bugge T So A Exacerbation of antigen-induced arthritis in urokinase-deficient mice J Clin Invest 1998 102 41 50 10.1172/JCI2312 9649555 PMC509063

[B7] Yang YH Carmeliet P Hamilton JA Tissue-type plasminogen activator deficiency exacerbates arthritis J Immunol 2001 167 1047 1052 11441114 10.4049/jimmunol.167.2.1047

[B8] Werd Z Alexander CM Proteinases and Matrix Degradation 1993 Philadelphia, PA: W.B. Saunders Co 248 268

[B9] Murphy G Atkinson S Ward R Gavrilovic J Reynolds JJ The role of plasminogen activators in the regulation of connective tissue metalloproteinases Ann N Y Acad Sci 1992 667 1 12 10.1111/j.1749-6632.1992.tb51590.x 1339240

[B10] Werb Z Mainardi CL Vater CA Harris ED Jr Endogenous activiation of latent collagenase by rheumatoid synovial cells. Evidence for a role of plasminogen activator N Engl J Med 1977 296 1017 1023 10.1056/NEJM197705052961801 66627

[B11] Campbell IK Last K Novak U Lund LR Hamilton JA Recombinant human interleukin-1 inhibits plasminogen activator inhibitor-1 (PAI-1) production by human articular cartilage and chondrocytes Biochem Biophys Res Commun 1991 174 251 257 10.1016/0006-291X(91)90513-7 1989604

[B12] Campbell IK Piccoli DS Butler DM Singleton DK Hamilton JA Recombinant human interleukin-1 stimulates human articular cartilage to undergo resorption and human chondrocytes to produce both tissue- and urokinase-type plasminogen activator Biochim Biophys Acta 1988 967 183 194 3142527 10.1016/0304-4165(88)90008-6

[B13] Hamilton JA Campbell IK Wojta J Cheung D Plasminogen activators and their inhibitors in arthritic disease Ann N Y Acad Sci 1992 667 87 100 10.1111/j.1749-6632.1992.tb51602.x 1309075

[B14] Hamilton JA Cheung D Filonzi EL Piccoli DS Wojta J Gallichio M McGrath K Last K Independent regulation of plasminogen activator inhibitor 2 and plasminogen activator inhibitor 1 in human synovial fibroblasts Arthritis Rheum 1992 35 1526 1534 10.1002/art.1780351217 1472130

[B15] Hamilton JA Hart PH Leizer T Vitti GF Campbell IK Regulation of plasminogen activator activity in arthritic joints J Rheumatol Suppl 1991 27 106 109 1902874

[B16] Hamilton JA Slywka J Stimulation of human synovial fibroblast plasminogen activator production by mononuclear cell supernatants J Immunol 1981 126 851 855 6161965

[B17] Hamilton JA Stanley ER Burgess AW Shadduck RK Stimulation of macrophage plasminogen activator activity by colony-stimulating factors J Cell Physiol 1980 103 435 445 10.1002/jcp.1041030309 6967488

[B18] Leizer T Clarris BJ Ash PE van Damme J Saklatvala J Hamilton JA Interleukin-1 beta and interleukin-1 alpha stimulate the plasminogen activator activity and prostaglandin E2 levels of human synovial cells Arthritis Rheum 1987 30 562 566 10.1002/art.1780300511 3109443

[B19] Ronday HK Smits HH Quax PH van der Pluijm G Lowik CW Breedveld FC Verheijen JH Bone matrix degradation by the plasminogen activation system. Possible mechanism of bone destruction in arthritis Br J Rheumatol 1997 36 9 15 10.1093/rheumatology/36.1.9 9117184

[B20] van der Laan WH Pap T Ronday HK Grimbergen JM Huisman LG TeKoppele JM Breedveld FC Gay RE Gay S Huizinga TW Verheijen JH Quax PH Cartilage degradation and invasion by rheumatoid synovial fibroblasts is inhibited by gene transfer of a cell surface-targeted plasmin inhibitor Arthritis Rheum 2000 43 1710 1718 10.1002/1529-0131(200008)43:8<1710::AID-ANR6>3.0.CO;2-Y 10943860

[B21] Li J Guo Y Holmdahl R Ny T Contrasting roles of plasminogen deficiency in different rheumatoid arthritis models Arthritis Rheum 2005 52 2541 2548 10.1002/art.21229 16052596

[B22] Kouskoff V Korganow AS Duchatelle V Degott C Benoist C Mathis D Organ-specific disease provoked by systemic autoimmunity Cell 1996 87 811 822 10.1016/S0092-8674(00)81989-3 8945509

[B23] Little CB Barai A Burkhardt D Smith SM Fosang AJ Werb Z Shah M Thompson EW Matrix metalloproteinase 13-deficient mice are resistant to osteoarthritic cartilage erosion but not chondrocyte hypertrophy or osteophyte development Arthritis Rheum 2009 60 3723 3733 10.1002/art.25002 19950295 PMC2832925

[B24] Mercuri FA Maciewicz RA Tart J Last K Fosang AJ Mutations in the interglobular domain of aggrecan alter matrix metalloproteinase and aggrecanase cleavage patterns. Evidence that matrix metalloproteinase cleavage interferes with aggrecanase activity J Biol Chem 2000 275 33038 33045 10.1074/jbc.275.42.33038 11032846

[B25] Lawlor KE Campbell IK O'Donnell K Wu L Wicks IP Molecular and cellular mediators of interleukin-1-dependent acute inflammatory arthritis Arthritis Rheum 2001 44 442 450 10.1002/1529-0131(200102)44:2<442::AID-ANR63>3.0.CO;2-M 11229476

[B26] Milner JM Rowan AD Cawston TE Young DA Metalloproteinase and inhibitor expression profiling of resorbing cartilage reveals pro-collagenase activation as a critical step for collagenolysis Arthritis Res Ther 2006 8 R142 10.1186/ar2034 16919164 PMC1779431

[B27] Moilanen M Sorsa T Stenman M Nyberg P Lindy O Vesterinen J Paju A Konttinen YT Stenman UH Salo T Tumor-associated trypsinogen-2 (trypsinogen-2) activates procollagenases (MMP-1, -8, -13) and stromelysin-1 (MMP-3) and degrades type I collagen Biochemistry 2003 42 5414 5420 10.1021/bi020582s 12731883

[B28] Birkedal-Hansen H Lin HY Birkedal-Hansen B Windsor LJ Pierson MC Degradation of collagen fibrils by live cells: role of expression and activation of procollagenase Matrix Suppl 1992 1 368 374 1480062

[B29] Milner JM Elliott SF Cawston TE Activation of procollagenases is a key control point in cartilage collagen degradation: interaction of serine and metalloproteinase pathways Arthritis Rheum 2001 44 2084 2096 10.1002/1529-0131(200109)44:9<2084::AID-ART359>3.0.CO;2-R 11592371

[B30] van Lent PL Holthuysen AE Sloetjes A Lubberts E van den Berg WB Local overexpression of adeno-viral IL-4 protects cartilage from metallo proteinase-induced destruction during immune complex-mediated arthritis by preventing activation of pro-MMPs Osteoarthritis Cartilage 2002 10 234 243 10.1053/joca.2001.0501 11869085

[B31] Jin T Tarkowski A Carmeliet P Bokarewa M Urokinase, a constitutive component of the inflamed synovial fluid, induces arthritis Arthritis Res Ther 2003 5 R9 R17 10.1186/ar606 12716448 PMC154426

[B32] Monach P Hattori K Huang H Hyatt E Morse J Nguyen L Ortiz-Lopez A Wu HJ Mathis D Benoist C The K/BxN mouse model of inflammatory arthritis: theory and practice Methods Mol Med 2007 136 269 282 full_text 17983155 10.1007/978-1-59745-402-5_20

[B33] Bugge TH Flick MJ Danton MJ Daugherty CC Romer J Dano K Carmeliet P Collen D Degen JL Urokinase-type plasminogen activator is effective in fibrin clearance in the absence of its receptor or tissue-type plasminogen activator Proc Natl Acad Sci USA 1996 93 5899 5904 10.1073/pnas.93.12.5899 8650190 PMC39159

[B34] Romer J Bugge TH Pyke C Lund LR Flick MJ Degen JL Dano K Impaired wound healing in mice with a disrupted plasminogen gene Nat Med 1996 2 287 292 10.1038/nm0396-287 8612226

[B35] Flick MJ LaJeunesse CM Talmage KE Witte DP Palumbo JS Pinkerton MD Thornton S Degen JL Fibrin(ogen) exacerbates inflammatory joint disease through a mechanism linked to the integrin alphaMbeta2 binding motif J Clin Invest 2007 117 3224 3235 10.1172/JCI30134 17932565 PMC2000806

[B36] Bryer SC Fantuzzi G Van Rooijen N Koh TJ Urokinase-type plasminogen activator plays essential roles in macrophage chemotaxis and skeletal muscle regeneration J Immunol 2008 180 1179 1188 18178858 10.4049/jimmunol.180.2.1179

[B37] Minami E Castellani C Malchodi L Deem J Bertko K Meznarich J Dishmon M Murry CE Stempien-Otero A The role of macrophage-derived urokinase plasminogen activator in myocardial infarct repair: Urokinase attenuates ventricular remodeling J Mol Cell Cardiol 2010 49 516 524 10.1016/j.yjmcc.2010.03.022 20380835 PMC3041515

[B38] Tortorella MD Malfait AM Deccico C Arner E The role of ADAM-TS4 (aggrecanase-1) and ADAM-TS5 (aggrecanase-2) in a model of cartilage degradation Osteoarthritis Cartilage 2001 9 539 552 10.1053/joca.2001.0427 11520168

[B39] Martel-Pelletier J McCollum R Fujimoto N Obata K Cloutier JM Pelletier JP Excess of metalloproteases over tissue inhibitor of metalloprotease may contribute to cartilage degradation in osteoarthritis and rheumatoid arthritis Lab Invest 1994 70 807 815 8015285

[B40] Kusano K Miyaura C Inada M Tamura T Ito A Nagase H Kamoi K Suda T Regulation of matrix metalloproteinases (MMP-2, -3, -9, and -13) by interleukin-1 and interleukin-6 in mouse calvaria: association of MMP induction with bone resorption Endocrinology 1998 139 1338 1345 10.1210/en.139.3.1338 9492070

[B41] van den Berg WB van de Loo FA van Lent PL Joosten LA Mechanisms of cartilage destruction in joint inflammation Agents Actions Suppl 1993 39 49 60 10.1007/BF01975714 8456643

[B42] Konig A Muhlbauer RC Fleisch H Tumor necrosis factor alpha and interleukin-1 stimulate bone resorption *in vivo *as measured by urinary [3H]tetracycline excretion from prelabeled mice J Bone Miner Res 1988 3 621 627 10.1002/jbmr.5650030607 3266953

[B43] Kang BS Park YG Cho JY Kim JK Lee TK Kim DW Gu YH Suzuki I Chang YC Kim CH Interleukin-1 and tumor necrosis factor-alpha induce collagenolysis and bone resorption by regulation of matrix metalloproteinase-2 in mouse calvarial bone cells Immunopharmacol Immunotoxicol 2003 25 347 364 10.1081/IPH-120024503 19180798

[B44] Cook AD Vlahos R Massa CM Braine EL Lenzo JC Turner AL Way KJ Hamilton JA The effect of tissue type-plasminogen activator deletion and associated fibrin(ogen) deposition on macrophage localization in peritoneal inflammation Thromb Haemost 2006 95 659 667 16601837

[B45] Wahl SM Arend WP Ross R The effect of complement depletion on wound healing Am J Pathol 1974 75 73 89 4825618 PMC1910809

